# Data‐Driven Engineering of Thermostable Collagen‐Mimetic Peptoid Triple Helices

**DOI:** 10.1002/marc.202500917

**Published:** 2026-02-24

**Authors:** Alex Berlaga, Renyu Zheng, Zeqian Zhang, Junhee Lee, Diya Gandhi, Chun‐Long Chen, Andrew L. Ferguson

**Affiliations:** ^1^ Department of Chemistry University of Chicago Chicago Illinois USA; ^2^ Department of Chemical Engineering University of Washington Seattle Washington USA; ^3^ Department of Chemistry University of Washington Seattle Washington USA; ^4^ Pritzker School of Molecular Engineering University of Chicago Chicago Illinois USA; ^5^ Physical Sciences Division Pacific Northwest National Laboratory Richland Washington USA; ^6^ Pritzker School of Molecular Engineering and Department of Chemistry University of Chicago Chicago Illinois USA

**Keywords:** active learning, biomaterials, collagen‐mimetic peptides, molecular design, molecular dynamics, peptoids

## Abstract

Collagen‐mimetic peptides (CMPs) are engineered molecules designed to replicate the triple‐helical structure of natural collagen. A repeating x–y‐Gly sequence is the defining motif of CMPs and is critical to their triple‐helical structure and stability. Substitutions to the residues occupying the x and y positions present a means to modulate the CMP structure and properties. Peptoid residues—N‐substituted glycine derivatives—present an attractive potential substitution due to their thermal stability, proteolytic resistance, biocompatibility, and diverse palette of non‐natural side chains, but also tend to introduce a high degree of backbone flexibility that can diminish the stability of the triple helix. In this work, we report a computational active learning cycle comprising molecular dynamics simulation, Gaussian process regression, and Bayesian optimization to computationally identify a number of promising peptoid substitutions predicted to stabilize the desired quaternary structure through side chain interactions and produce stable peptoid‐based collagen‐like triple helices. To experimentally test the computational predictions, a top candidate identified by the screen was synthesized and imaged using scanning electron microscopy to resolve fibril‐like bundles consistent with collagen‐like triple helices. This work predicts a number of CMP peptoid substitutions capable of forming stable triple‐helical structures, presents a generalizable design strategy for engineering desired peptoid structures, and opens new avenues for the design of peptoid‐based biomimetic materials.

## Graphical abstract

Data‐driven active learning discovery of collagen‐mimetic peptoids capable of forming a thermostable collagen‐like triple helix using computational screening followed by experimental validation.

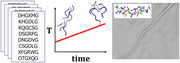

## 1 Introduction

Collagen, the primary structural protein in connective tissues, is a natural biopolymer of biomedical interest due to its unique triple‐helical tertiary structure and wide‐ranging applications [1, 2, 3, 4], including wound and tissue repair [1], drug delivery [5], and cosmetics [6]. The defining sequence motif of natural collagen is its repeating x–y‐Gly amino acid triplet, where the glycine (Gly) residue is known to be critical for the close packing of the triple helix, while *x* and *y* can admit substitutions for various amino acid residues [3, 7, 8]. The individual strands form a polyproline‐II helix‐like secondary structure [9] and the primary stabilizing force in the triple helix is the hydrogen bonds between the amide ─N─H hydrogen in the glycine and ─C═O carbonyl carbon groups on corresponding residues in a neighboring strand [10]. In humans, approximately 33% of the *x* and *y* positions are occupied by proline (Pro) and hydroxyproline (Hyp) residues [11]. The Pro and Hyp residues provide rigidity in the backbone and reduce the entropic cost of folding into the polyproline‐II helix‐like precursors that constitute the collagen triple helix [3]. Variations in the *x* and *y* residues allow for the diversity observed among collagen types, influencing properties such as flexibility and binding specificity [12]. Understanding the sequence–structure relationship in collagen has guided the design of synthetic collagen‐mimetic peptides (CMPs) as engineered analogs with the potential to introduce novel structure and/or function [10, 13, 14]. For instance, the RGD (Arg–Gly–Asp) motif has been used to engineer CMPs that promote fibroblast attachment and migration with applications in accelerated wound healing [12, 15], and the EOG (Glu–Hyp–Gly) motif is known to give CMPs the propensity to form nanospheres with potential applications in the delivery of therapeutics [16].

Recent advancements in collagen research have explored the incorporation of unnatural amino acids to further enhance the properties of CMPs, especially the stability of the triple helix quaternary structure that can enable these molecules to retain their structure at high temperatures or in denaturing environments [3, 4, 10]. For example, fluoroproline and alkylated prolines have been shown to improve the thermal stability of CMPs by creating induced dipole–dipole interactions that strengthen interactions between strands in the triple helix [17]. The incorporation of peptoid residues—N‐substituted glycine derivatives—as opposed to amino acid residues has also garnered recent interest for their ability to provide proteolytic resistance and offer a diverse palette of non‐natural side chains while maintaining biocompatibility [18, 19]. Peptoids differ from peptides in that the residue side chains are connected to the backbone via the nitrogen atom as opposed to the α‐carbon. This seemingly small change leads to a number of altered properties, including the elimination of the α‐carbon chiral center, ─N─H hydrogen bond donor, and planar peptide bond, and the accessibility of both the cis and trans states of the backbone ω dihedral [20, 21, 22]. Despite the promise of peptoids in CMP engineering, achieving a stable triple helix primarily using peptoid residues presents a challenge since, while the cis configuration of the ω backbone dihedral is typically sterically forbidden to peptide residues by the strongly planar trans peptide bond, the accessibility of the cis and trans states in peptoid residues opens additional conformational states that induce significantly more backbone flexibility and entropically destabilize the collagen triple helix relative to the dissociated strands [20, 22, 23, 24, 25, 26, 27, 28, 29].

In this work, we introduce non‐natural peptoid residues at both the *x* and *y* positions of the *x*–*y*‐Gly collagen repeat to engineer novel collagen mimetic peptoids to realize the proteolytic resistance and chemical versatility of peptoids with the functional benefits of collagen‐like architectures. Interestingly, the amino acid residues glycine (Gly), proline (Pro), and hydroxyproline (Hyp) prevalent in natural collagen are themselves all identical in both peptide and peptoid forms because their side chains are hydrogen, pyrrolidine, and hydroxypyrollidine rings, respectively. As such, while Gly–Pro–Hyp may formally be regarded as a peptoid, it is a very irregular one, since proline and hydroxyproline are exceptional in that their side chains are covalently bonded to both the α‐carbon and the backbone nitrogen, strongly restricting local conformational flexibility of the backbone and promoting preorganization of the backbone into extended polyproline‐II‐like helices that constitute the collagen triple helix [18]. The rigidity imparted by the Pro and Hyp residues stands in contrast to typical peptoid residues in which the side chains are connected to the backbone only through the backbone nitrogen and possess a high degree of backbone flexibility due to the accessibility of both the cis and trans states of the ω dihedral. In this study, we consider only the latter class of “normal” peptoid residues and seek to compensate for the entropic penalty disfavoring the collagen triple helix quaternary structure attending the elevated backbone flexibility by engineering the chemistry of the peptoid side chains to introduce intra‐ and inter‐molecular interactions that thermodynamically favor the triple helical state. Henceforward, we will overload the acronym CMP to stand for both collagen‐mimetic peptides and collagen‐mimetic peptoids, and take care to distinguish one from the other as necessary.

Our study builds upon the foundational experimental and computational contributions of a number of prior studies. Goodman et al. [30] demonstrated that a peptoid–peptide hybrid—specifically with NLeu substituted into the *y* position to form a Pro–NLeu–Gly collagen repeat—produces a triple helical structure that is comparably stable to native collagen. Building on this, Kessler et al. [19] evaluated the stabilizing and destabilizing effects of various peptoid residues within an *x*‐Hyp–Gly repeat upon the triple helix to achieve an expanded library of potential collagen‐mimetic structures. More recently, Qiu et al. [18] showed that although collagen triple helices are composed entirely of trans peptide bonds, introducing a cis‐promoting residue in place of proline within a single repeat of a Pro–Hyp–Gly motif can, somewhat unexpectedly, actually enhance the stability of the triple helix. Together, these studies establish important demonstrations and design principles for incorporating peptoids into CMPs. In all cases, however, the stability of the CMP triple helices benefits, as in the case of natural collagen, from the backbone rigidity provided by the repetitive pyrrolidine and/or hydroxypyrollidine rings present, respectively, in the repetitive Pro and/or Hyp residues. In this work, we seek to engineer CMPs composed of x‐y‐Gly motifs in which both x and y are typical peptoids with side chains attached only through the backbone nitrogen.

Advances in computational modeling have also advanced efforts to understand and engineer CMPs, particularly in informing the sequence‐specific formation and stability of the collagen triple helix. Molecular dynamics (MD) simulations have provided insights into the folding kinetics, thermal stability, and critical stabilizing interactions of the collagen triple helix in natural collagen and CMPs [31, 32]. Taylor et al. [33] studied x‐y‐Gly motifs, where x and y are modified prolines, to expose the critical role of side‐chain interactions and water‐mediated hydrogen bonds. Fallas and Hartgerink [13] devised computational scoring functions to identify sequences likely to form inter‐chain salt bridges that are likely to stabilize a triple helix. Molecular simulation is valuable in both providing fundamental understanding of the sequence–structure–stability relationship for rational design and also in prospectively predicting the effects of alterations to the sequence prior to experimental synthesis and characterization. The latter modality is particularly helpful in the context of peptoid‐dominant CMP design due to the exceedingly large palette of possible non‐natural peptoid side chains and the experimental labor associated with the synthesis of complex peptoid side chain chemistries [34, 35].

In this work, we combine MD simulations with Bayesian optimization (BO) to discover x–y‐Gly peptoid sequences capable of forming collagen triple helices with stabilities comparable to or surpassing natural collagen. Specifically, we employ temperature‐ramping MD simulations within a high‐throughput virtual screening (HTVS) pipeline to computationally assess the stability of collagen‐like triple helices formed from sequence‐defined peptoid‐based CMPs. The calculated thermodynamic stabilities of each sequence were used to train a Gaussian process regression (GPR) surrogate model that we use to predict the stability of as‐yet unexplored sequences and we prioritize top candidates for subsequent simulations using BO. Taken together, these three components form a virtuous design‐build‐test‐learn (DBTL) cycle: with each iteration, the MD simulations explore more regions of sequence space and furnish more data with which to refine the GPR surrogate model while the GPR model and BO guide the MD simulations towards the most promising sequences. Similar simulation‐based active learning loops have been successfully employed for a variety of molecular discovery and design tasks, including applications to optically and electronically active π‐conjugated peptides [36], functional drug‐like molecules [37], and thermostable metal alloys [38]. Commencing from a candidate library of 10^4^ possible 18‐residue peptoid sequences (x1‐y1‐G‐x2‐y2‐G)_3_, our computational active learning screen converges after 24 cycles and direct simulation of only 218 sequences (∼2% of the design space) to identify five peptoid‐based CMPs with predicted stabilities on par with or surpassing a natural collagen variant with a Pro–Pro–Gly triplet repeat as evaluated by free energy calculations of helix denaturation. A post hoc compositional analysis of the active learning campaign exposes a number of rational design principles, including a critical role for charged and light hydrophobic residues to promote stabilizing inter‐strand interactions and compensate for the elevated backbone flexibility. We experimentally tested the computational predictions by synthesizing a top candidate sequence identified by the screen and resolved fibril‐like bundles consistent with collagen‐like triple helices using scanning electron microscopy (SEM) imaging. Taken together, this work predicts a number of CMP peptoid substitutions capable of forming stable triple‐helical structures and presents a generalizable design strategy for engineering a desired quaternary structure into peptoid‐based biomimetic supramolecular assemblies.

## 2 Methods

**FIGURE 1:**
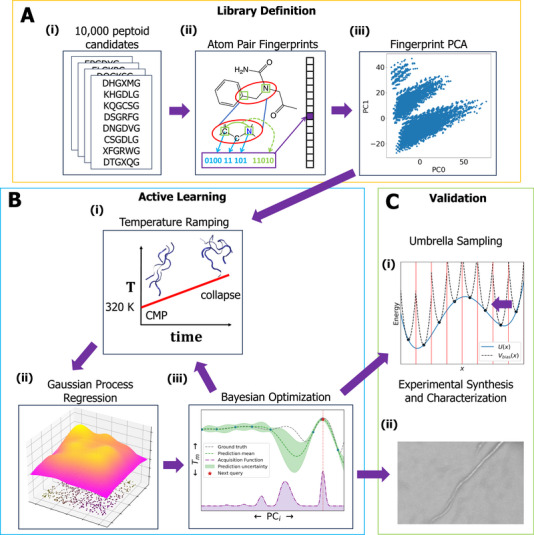
Schematic illustration of the computational discovery pipeline for peptoid‐based CMPs capable of forming a thermostable collagen triple helix. (A) Definition of the x‐y‐Gly peptoid‐only CMP sequence library. (i) The candidate space is defined by 18‐residue peptoid sequences comprising three repeating hexamer subunits x1‐y1‐Gly‐x2‐y2‐Gly, where x1 and x2 admit one of five charged peptoid residues (NLys, Nae, NCys, NAsp, NGlu), y1 and y2 admit one of twenty peptoid residues (NLys, Nae, NCys, NAsp, NGlu, NHis, NLeu, NMet, NPhe, NTyr, NIle, NThr, NVal, NTrp, Nspe, Ntbu, Nbrpm, Nbrpe, NAsn, NGln), and the linear chains are terminated by an acetyl N‐cap and an amino C‐cap. The resulting combinatorial space of repeat units spans $5^{2}$ × $20^{2}$ = 10 000 candidates. (ii) Each candidate is represented to the surrogate machine learning model as a 2048‐bit atom pair fingerprint. (iii) The active learning is conducted over an embedding of the 10 000 candidates into the top 10 principal components (PCs) of the library of atom pair fingerprints. (B) The design‐build‐test‐learn active learning cycle. (i) Molecular dynamics (MD) simulations of a preassembled collagen triple helix are subjected to temperature ramping to assess the relative stability of candidate sequences by providing an approximate estimate of the triple helix melting temperature $T_{m}$. (ii) Gaussian process regression (GPR) surrogate models are trained to predict the calculated $T_{m}$ values as a function of the coordinates of the candidate CMP sequence in the 10‐dimensional PC embedding, and permit inexpensive extrapolative predictions of $T_{m}$ for candidate sequences in the library that have not yet been simulated. (iii) BO is used to interrogate the predictions of the GPR trained over all calculations conducted to date to prioritize the next sequences to subject to MD simulations to close the active learning loop. (C) Computational and experimental validation of the top candidates identified in the active learning cycle. (i) Enhanced sampling free energy calculations employing umbrella sampling (US) are used to estimate the free energy difference between the native collagen triple helix and its melted state as a more rigorous, but computationally expensive, estimation of the thermodynamic stability of the triple helix. (ii) Experimental synthesis and characterization of a top candidate using scanning electron microscopy (SEM) resolves fibril‐like bundles consistent with collagen‐like triple helices.

We develop and deploy an active learning framework for the discovery of peptoid‐based CMPs capable of forming a thermostable collagen triple helix. We begin with a rationally designed library of 10 000 18‐residue peptoid sequences composed of x–y‐Gly motifs informed by established guidelines for stabilizing triple‐helical tertiary structures [8, 10, 12, 19] and employ an atom‐pair‐based featurization of each sequence based on atom pair fingerprints (Figure 1A). We develop an active learning strategy using temperature‐ramping MD to estimate melting temperatures, GPR surrogate models linking sequence to stability, and BO to guide efficient traversal of sequence space (Figure 1B). We computationally validate the top sequences identified by our active learning cycle using enhanced sampling MD calculations employing umbrella sampling to evaluate the thermodynamic stability of the collagen triple helix, and then subject our top design to experimental synthesis and characterization (Figure 1C). In the following sections, we provide details of each component of the active learning cycle. Jupyter notebooks implementing all computational components of the active learning workflow are available at https://github.com/Ferg‐Lab/cmp.

### 2.1 Peptoid Design Space

**FIGURE 2:**
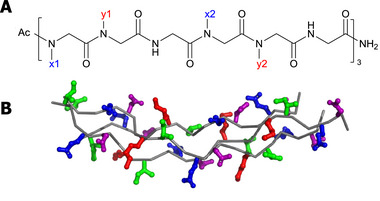
Peptoid design space and target collagen triple helix. (A) Chemical structure of an 18‐residue peptoid comprising three repeating hexamer subunits (x1‐y1‐Gly‐x2‐y2‐Gly)_3_ terminated by an acetyl N‐cap and an amino C‐cap. The x1 and x2 positions are selected to be one of five charged residues that promote stabilization of a polyproline‐II helix‐like structure in a single strand and promote favorable inter‐strand electrostatic interactions with the partial charges on the strongly dipolar carbonyl group. The y1 and y2 positions are selected to be one of a larger set of twenty peptoid residues identified as not to impair stability of the collagen triple helix relative to Pro. The particular peptoid residues admitted in the x and y positions are listed in Table 1. The family of 18‐mer hexamer repeat peptoids constitutes a design space of $5^{2}$ × $20^{2}$ = 10 000 candidate sequences. (B) Space filling structure of three 18‐mer strands with sequence (NLys–NIle–Gly–NGlu–NAsn–Gly)_3_ in a collagen triple helix quaternary structure. The peptoid backbones are represented in gray, the NLys side chains in red, the NIle side chains in green, the NGlu side chains in blue, and the NAsn side chains in purple.

We define as our design space of peptoid‐based CMPs the family of 18‐residue peptoids comprising three repeating hexamer subunits (x1‐y1‐Gly‐x2‐y2‐Gly)_3_ terminated by an acetyl N‐cap and an amino C‐cap (Figure 1A(i), Figure 2A). This design space preserves the fundamental x–y‐Gly triplet of natural collagen, but we select a hexamer rather than a trimer as our fundamental unit. The hexamer retains the collagen‐like Gly every third residue yet, unlike the canonical (x–y‐Gly) trimer, provides four independently tunable positions (x1, x2, y1, y2) that permit a broader potential for modulation of charge, hydrophobicity, and hydrogen‐bond donors. We also choose to consider 18‐mers as a relatively short sequence that is amenable to efficient high‐throughput molecular simulation within our computational active learning cycle. As in natural collagen and peptide CMPs [39], we choose to maintain Gly residues every three positions to permit close approach of the strands and promote hydrogen bonding between the ─N─H amide hydrogen in the glycine side chain and ─C═O carbonyl oxygen. For the remaining residues in the six‐residue repeat, we apply heuristic restrictions adopted from studies of natural collagen and CMP. The individual strands comprising the collagen triple helix adopt a polyproline‐II helix‐like secondary structure [9]. In peptoid chains, many polyproline‐II helix‐like structures have the property that at least every third residue is charged near physiological pH [40, 41, 42, 43]. This observation motivated us to narrow our design space by enforcing a charged peptoid residue to occupy the x1 and x2 positions to promote stabilization of single strands into polyproline‐II helix‐like structures and also promote inter‐strand electrostatic interactions between these formally charged residues on one strand and, potentially, oppositely charged residues on another, as well as the partial charges on the strongly dipolar carbonyl groups. The chemical palette of possible peptoid side chains is, formally, infinite, but we are motivated to restrict our selection of charged residues to five residues (NLys, Nae, NCys, NAsp, NGlu) that are charged at or near physiological pH and are supported within the recently developed MoSiC‐CGenFF‐NTOID all‐atom peptoid force field that we use to conduct the MD simulations reported in this work [44]. While we include NCys among the “charged” residues because ∼11% of its side chains are deprotonated at physiological pH, we simulate it in the neutral state (and label it as neutral in further analysis); however, as we later show, this inclusion does not have any effect on our analysis because NCys‐containing sequences generally emerge among the least stable triple‐helical peptoids, and its inclusion effectively serves as a negative control for the importance of charged residues in these positions. Although supported by the force field, we choose to exclude NArg from this group due to the requirement of a complicated orthogonal protection method for the synthesis of NArg‐containing peptoids [45]. For the y1 and y2 positions, we permit the substitution of the twenty peptoid residues supported by the force field (NLys, Nae, NCys, NAsp, NGlu, NHis, NLeu, NMet, NPhe, NTyr, NIle, NThr, NVal, NTrp, Nspe, Ntbu, Nbrpm, Nbrpe, NAsn, NGln), while excluding the NAla, NSer, and NEme residues shown by Kessler et al. to weaken triple‐helix stability relative to proline [19], and all chlorine‐, fluorine‐, or iodine‐containing halogenated residues, which are less accessible synthetically than their brominated counterparts. By permitting the (x1, x2) and (y1, y2) residues within the hexamer repeat to be independently selected from these groups of 5 and 20 possibilities, our design space comprises a total of $5^{2}$ × $20^{2}$ = 10 000 candidate sequences. The chemical structure and space‐filling model of the corresponding collagen triple helix of one such candidate (NLys–NIle–Gly–NGlu–NAsn–Gly)_3_ is presented in Figure 2B as an illustrative example. In Table 1, we present a list of the 20 possible peptoid residues considered in this work providing a list of their three letter codes, one letter codes, and chemical structures.

**TABLE 1:** Three letter codes, one letter codes, name, and charge of the 22 peptoid residues involved in this work.

Residue	Chemical name	1‐letter code	Charge (pH 7)	Positions
NCys	*N*‐(2‐mercaptoethyl)glycine	C	0*	x, y
NAsp	*N*‐(2‐carboxyethyl)glycine	D	−1	x, y
NGlu	*N*‐(3‐carboxypropyl)glycine	E	−1	x, y
NPhe	*N*‐benzylglycine	F	0	y
Gly	glycine (no side chain)	G	0	—
NHis	*N*‐(4‐imidazolyl)ethylglycine	H	0	y
NIle	*N*‐*sec*‐butylglycine	I	0	y
NLys	*N*‐(4‐aminobutyl)glycine	K	+1	x, y
NLeu	*N*‐isobutylglycine	L	0	y
NMet	*N*‐(2‐methylthioethyl)glycine	M	0	y
NAsn	*N*‐(2‐carbamoylethyl)glycine	N	0	y
Pro	proline (pyrrolidine ring)	P	0	—
NGln	*N*‐(3‐carbamoylpropyl)glycine	Q	0	y
NThr	*N*‐(1‐hydroxyethyl)glycine	T	0	y
NVal	*N*‐isopropylglycine	V	0	y
NTrp	*N*‐(indol‐3‐ylmethyl)glycine	W	0	y
Nae	*N*‐(2‐aminoethyl)glycine	X	+1	x, y
NTyr	*N*‐(*p*‐hydroxybenzyl)glycine	Y	0	y
Nspe	*N*‐(*S*‐1‐phenylethyl)glycine	Z	0	y
Ntbu	*N*‐*tert*‐butylglycine	1	0	y
Nbrpm	*N*‐(4‐bromomethylbenzyl)glycine	3	0	y
Nbrpe	*N*‐(4‐bromoethylbenzyl)glycine	4	0	y

* The nominal ‐SH (same thing) group $pK_{a}$ = 8.3 dictates that at physiological pH the side chain is largely neutral (∼11% deprotonated).

*Note*: The particular residues admitted for incorporation into the (x1, x2) and (y1, y2) positions within the (x1–y1‐Gly‐x2–y2‐Gly) hexamer repeat are indicated in Column 5 as “x” and “y”, respectively.

### 2.2 Peptoid Sequence Featurization

We next define a featurization of the 10 000 CMP candidates in order to represent them to our GPR surrogate model within the active learning cycle. Multiple choices of molecular featurizations are possible, including one‐hot encodings, molecular graph representations [46], fingerprint‐based vectors [47, 48, 49], and low‐dimensional embeddings learned by unsupervised learning [36, 37]. In this work, we elect to employ atom pair fingerprints as a representation of a molecule based on the presence of specific pairs of atoms and their relative positions [48] (Figure 1A(ii)), and which has previously been used to featurize peptides [50]. We use RDKit [51] to generate hashed atom‐pair fingerprints for the hexamer repeat that defines each of our 10 000 CMP candidates. For every unordered pair of heavy atoms within a cutoff of five covalent bonds, RDKit records the element, charge, hybridization, and ring status of each atom together with the topological separation of the pair. The features are hashed into a 2048‐bit vector that is long enough to minimize collisions yet compact enough to avoid excessive sparsity. Formally, this featurization constitutes a 2D atom pair fingerprint that encodes information on the bonded atom types and covalent connectivity within the chemical structure of the molecule, but which neglects 3D information on the spatial proximity of atoms within the tertiary (and quaternary) structure. The calculation of 3D structural fingerprints would require access to the molecular structure of each CMP, which, for highly flexible molecules such as these, is expected to comprise an ensemble of conformations as opposed to a single relatively rigid structure as tends to be the case for small molecules. Adequate sampling of the conformational ensemble is expected to present a challenge for these relatively large molecules and, from a pragmatic perspective, entail a very high computational cost to do so for the 10 000 CMP candidates in our library. As such, we implicitly assume that 2D molecular fingerprints can provide a sufficiently rich structural featurization to construct predictive surrogate models of CMP stability as a function of sequence. We provide below a posteriori support for this hypothesis via the success of the active learning screen in identifying top CMP candidates.

The 2048‐dimensional atom pair bit vectors provide a rich molecular featurization, but training of GPR models capable of extrapolating over the candidate space without overfitting and facilitating navigation of the search space using BO typically proceeds better in low‐dimensional, structured spaces [52]. As such, we sought to reduce the dimensionality of the atom pair featurization by applying principal components analysis (PCA) to the 10 000‐by‐2048 matrix representing the atom pair featurization of all CMP candidates to identify a smaller number of collective variables corresponding to high variance directions within the feature space [53]. The top 10 principal components (PCs) were found to capture 74.1% of the variance in the data, permitting us to construct a 10D feature space in which to train the GPR surrogate model (Figure 1A(iii)). Furthermore, the leading PCs were found to possess strong physical interpretability consistent with chemical intuition and a strong structuring of the candidate space within the PC embedding (Figure S1). For example, the top principal component was correlated with the number of NLys residues in the first and fourth positions and the number of Nspe, Nbrpe, or Nbrpm residues in the second and fifth positions. Interestingly, these precise residues may uniquely interact as electrophiles in n–$\pi^{*}$ or nucleophile–electrophile interactions with the peptoid carbonyl oxygen. The second principal component was correlated with the hexamer molecular weight, while the third was correlated with the number of negatively charged residues.

### 2.3 Non‐Equilibrium Melting Temperature Estimate of Triple Helix Stability

We estimated the stability of the collagen triple helixes formed by various candidate CMPs by conducting temperature ramping molecular dynamics (MD) simulations to estimate the melting temperature $T_{m}$ at which the quaternary structure is compromised and the helix denatures (Figure 1B(i)). We eschew more rigorous equilibrium free energy estimations of stability within the active learning loop for a cheaper, more approximate non‐equilibrium estimation procedure in order to increase the throughput of our computational screen. For the same reason, employ a relatively fast 1 K/ns temperature ramp rate that is anticipated to lead to potential hysteresis and overestimation of $T_{m}$ but, nevertheless, provide a reliable measure of relative stability of the sequences. As we discuss below, the non‐equilibrium $T_{m}$ values do indeed show strong correlation with the free energy difference between the native triple helix and its melted state computed by more computationally expensive enhanced sampling equilibrium free energy calculations (Section 2.6), thereby providing post hoc support for the $T_{m}$ estimates employed in our active learning cycle.

All‐atom MD simulations of the 18‐mer peptoid sequences were conducted using GROMACS version 2023.1 [54] patched with the PLUMED 2.10.0 libraries [55, 56]. Peptoid sequences were modeled using the MoSiC‐CGenFF‐NTOID force field [44] that is based on Weiser and Santiso's CGenFF‐NTOID reparameterization [57] of the CHARMM36 force field [58, 59, 60, 61]. Initial single chain conformations were generated using a peptoid structure generator developed in our previous work [44]. Commencing from a collagen triple helix structure adapted from PDB ID 1K6F [62] and composed of three (Gly)_18_ strands in polyproline‐II helix‐like conformations, we constructed each particular sequence of interest by grafting the necessary side chains onto each residue by substituting out the Gly hydrogen. Simulations were initialized from the natural right‐handed collagen triple helix, but the inherently achiral nature of peptoid chains means that the left‐ and right‐handed helices are expected to be equally stable. Each triple helix was then placed into a 7.2 ×7.2 ×7.2 ${\mathrm{nm}}^{3}$ cubic box implementing periodic boundary conditions in all dimensions and subjected to steepest descent gas phase energy minimization until the maximum force was below 1000 kJ/mol.nm or a maximum of 200 000 energy minimization steps was reached. Systems were solvated in SPC/E water [63] to a density of 1 g/cm^3^, corresponding to conditions of standard temperature and pressure, resulting in the insertion of ∼12 000 water molecules. In the case of charged systems, compensatory ${\mathrm{Na}}^{+}$ or ${\mathrm{Cl}}^{-}$ ions were added as necessary to neutralize the net charge. Electrostatics were treated using Particle–Mesh–Ewald [64] with a real‐space cutoff of 1.2 nm and a Fourier grid spacing of 0.06 nm that was subsequently optimized during runtime. Lennard–Jones interactions were smoothly shifted to zero at a cutoff of 1.2 nm. Initial velocities were assigned from a Maxwell‐Boltzmann distribution at 320 K. The classical equations of motion were integrated using a 2 fs time step under the leap‐frog algorithm [65]. Covalent bonds involving hydrogen atoms were fixed using the LINCS algorithm [66]. Center‐of‐mass (COM) translation and rotation were removed every 1 ps. A Verlet cutoff scheme was used with a neighbor list updated every 15 time steps. Simulation trajectories were written to disk at a period of 20 ps. Equilibration was first performed for 200 ps in the NVT ensemble at 320 K using a Bussi–Parrinello–Donadio velocity rescaling thermostat [67] with a time constant of 0.1 ps. This was followed by 200 ps of equilibration in the NPT ensemble at 320 K and 1 bar with a 1 fs time step, a Bussi–Parrinello–Donadio velocity rescaling thermostat [67] with a coupling time constant of 0.1 ps, and an isotropic Berendsen barostat with a coupling time constant of 1 ps and an isothermal compressibility of 4.5$\times 10^{-5}$ ${\mathrm{bar}}^{-1}$. Production runs were conducted in the NPT ensemble at 1 bar with a 2 fs time step employing a Nosé–Hoover thermostat [68] with a time constant of 1 ps and an isotropic Parinello–Rahman barostat [69] with a time constant of 1 ps and an isothermal compressibility of 4.5$\times 10^{-5}$ ${\mathrm{bar}}^{-1}$. The temperature in the production runs was linearly ramped from 320 K at a rate of 1 K/ns. Simulations were terminated and the melting temperature $T_{m}$ defined as the temperature at which the radius of gyration of the triple helix first dips below $R_{g}$ = 1.25 nm for at least 200 ps or the central α‐carbons of any pair of strands dissociates to a distance of at least 2 nm for 200 ps. These two thresholds account for the two observed predominant mechanisms of triple helix melting: collapse and dissociation. Simulations were conducted on a single NVIDIA V100 or A100 GPU to achieve execution speeds of ∼100‐300 ns/day. Uncertainties in $T_{m}$ estimates were quantified by conducting six replicas for each peptoid sequence and computing the standard error over the replicas.

**FIGURE 3:**
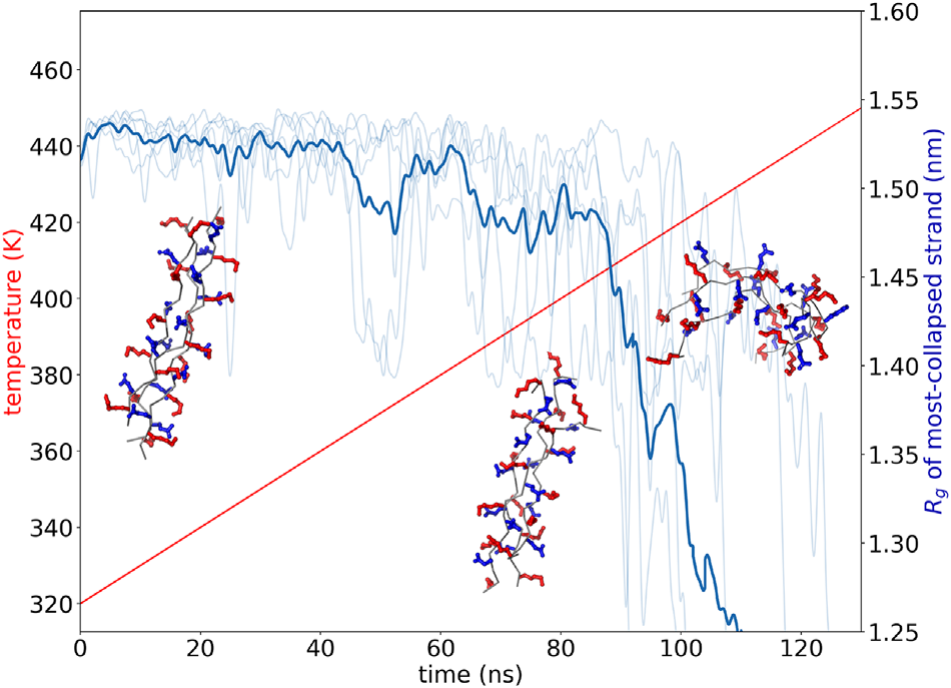
Illustration of the temperature ramping protocol and non‐equilibrium estimation of $T_{m}$ for the (NGlu–NLys–Gly–NGlu–NLys–Gly)_3_ CMP candidate. The initial triple helix conformation (left structure) remains stable until approximately 80 ns and 400 K, at which point the termini begin to fray (middle structure) and the quaternary structure undergoes denaturation and collapse (right structure). The linear temperature ramp (red line, left axis) proceeds at a ramp rate of 1 K/ns. Denaturation is tracked by following the radius of gyration $R_{g}$ of the most collapsed of the three strands (blue lines, right axis). The light blue lines report the smallest $R_{g}$ of the three strands in six replicate runs and the dark blue lines report the mean. We identify $T_{m}$ as the temperature at which the radius of gyration of the triple helix first dips below $R_{g}$ = 1.25 nm for at least 200 ps or the central α‐carbons of any pair of strands dissociates to a distance of at least 2 nm for 200 ps.

We present in Figure 3 an illustration of the temperature ramping protocol and non‐equilibrium estimation of $T_{m}$ for the (NGlu‐NLys‐Gly‐NGlu‐NLys‐Gly)_3_ peptoid candidate. Commencing from a collagen triple helix conformation at t = 0 ns and T = 320 K, the quaternary complex remains stable for the first approximately 80 ns until a temperature of approximately 400 K is reached, at which point the complex frays at the termini and then rapidly undergoes denaturation and catastrophic collapse. We find the radius of gyration of the most collapsed of the three strands (i.e., the strand with the smallest $R_{g}$) to be a simple and useful metric with which to track the quaternary structure of the three‐strand complex over the course of the temperature ramp. Frequently, we see the strands exhibit folding or collapse into a coil‐like conformation that are reminiscent of the collapse patterns observed by Bozec et al. [70] in atomic force microscopy characterization of temperature‐induced gelatinization of collagen [71, 72].

### 2.4 Gaussian Process Regression Surrogate Model

In each round of the active learning cycle, we train a Gaussian process regression (GPR) model to predict $T_{m}$ of a particular CMP candidate as a function of its coordinates in the 10D embedding into the leading PCs of the atom pair fingerprints (Figure 1B(ii)). The model is trained over all simulation data collected thus far and serves as an inexpensive surrogate model to predict $T_{m}$ for all remaining members of the candidate library. We favor GPR over other regression models for its non‐parametric and non‐linear nature that can flexibly model complex molecular property relationships and intrinsic uncertainty estimates that facilitate interfacing with BO [73, 74]. We employ the popular and flexible radial basis function (RBF) kernel [75] and optimize the length scale hyperparameter for each of the ten PC dimensions by marginal log‐likelihood maximization during each pass through the active learning loop. Uncertainties in the calculated $T_{m}$ values passed to the model are estimated from standard errors over the six replicate temperature ramp simulations and are incorporated into GPR training by adding a diagonal matrix of these standard errors to the kernel matrix for the training data during the calculation of the log‐likelihood [76]. Models are constructed as SingleTaskGP objects within BoTorch [77, 78] and optimization of the RBF length scale hyperparameters is implemented with GPyTorch [78, 79] using the Adam optimizer [80]. Model training requires ∼15 s on a single NVIDIA V100 GPU. The trained models are conditioned on all previous training data and can predict $T_{m}$ and an associated uncertainty for any location within the 10D PC embedding. Deployment of the trained model over all remaining candidates within the library of 10 000 candidate CMPs during each active learning cycle requires ∼0.1 s on a single NVIDIA V100 GPU.

### 2.5 Bayesian Optimization

**FIGURE 4:**
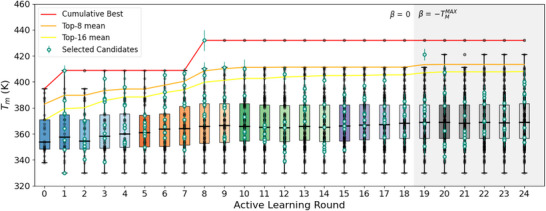
Results of the active learning screen for thermostable peptoid‐based CMP candidates. Box‐and‐whisker plots of calculated CMP melting temperatures $T_{m}$ in each round of the campaign. Grey points represent CMP candidates selected in previous rounds of the campaign, and green points represent those selected to be considered in the present round. The red curve represents the cumulative best $T_{m}$ of any candidate identified thus far in the campaign. The orange and yellow lines represent the mean $T_{m}$ of, respectively, the top‐8 and top‐16 candidates identified thus far. During the first 18 rounds, the batched BO is operated in a balanced exploit/explore modality by employing a qEI trade‐off parameter of β = 0. In the final six rounds, we operate in a pure exploit strategy by employing β = $-T_{m}^{\text{MAX}}$, where $T_{m}^{\text{MAX}}$ is the maximum $T_{m}$ value observed over all candidates to date. The top performing sequence, (EKGEKG)_3_ was identified in Round 8 and possesses a $T_{m}$ = (432 ± 8) K.

The final component of the active learning cycle employs BO to interrogate the predictions of the trained GPR model and prioritize candidates for the next round of MD simulation and computational $T_{m}$ evaluation according to an acquisition function (Figure 1B(iii)) [81, 82, 83, 84]. We elect to employ the q‐Expected Improvement (qEI) acqusition function, which extends the concept of expected improvement (EI) to batched selections of q≥1 candidates in each round [85]. Our available compute resources permitted us to conduct simultaneous evaluation of up to eight candidate sequences, motivating us to set q=8. Throughout our active learning campaign, we adjust qEI's tradeoff parameter β to regulate the balance between exploration and exploitation in the navigation of CMP candidate space. During the first 18 rounds, we set β = 0 corresponding to a balanced exploration and exploitation strategy analogous to the standard expected improvement acquisition function [85] in order to promote a global search of candidate space. After we ceased to see any additional improvements in the mean $T_{m}$ of the top eight candidates, we set β = $-T_{m}^{\text{MAX}}$, where $T_{m}^{\text{MAX}}$ is the maximum $T_{m}$ value observed over all candidates to date, to induce qEI to operate in a pure exploitation modality for an additional six rounds of the active learning cycle. We terminated the active learning cycles after observing no further improvements in the mean $T_{m}$ of the top‐8 candidates for four consecutive rounds. This two‐phase strategy follows up an early global search with late greedy exploitation to initially cast a wide net over candidate space and then, once that search ceases to show further improvements, focus on extracting the top performers (Figure 4). We implement our BO selection using BoTorch [77]. The candidates selected in each round together with the $T_{m}$ values estimated in each of the six replicate simulations are reported graphically in Figure S2 and in machine readable form in the csv file provided as Table S1.

### 2.6 Enhanced Sampling Free Energy Estimate of Triple Helix Stability

The active learning cycle evaluated the triple helix stability for the CMP candidates based on a computationally efficient approximate estimation of the melting temperature $T_{m}$ to support a high throughput virtual screen (Section 2.3). The top candidates identified after termination of the active learning search were then subjected to more rigorous stability estimates employing enhanced sampling free energy calculations. Specifically, we conducted umbrella sampling [86] to estimate the free energy difference ΔF between the native collagen triple helix configuration and the melted disordered aggregate. We define a reaction coordinate connecting these two states by performing time‐lagged independent component analysis (TICA) [87] of the temperature ramping calculations used to perform our approximate non‐equilibrium estimation of the melting temperature (Section 2.3) and extracting the dominant time‐lagged independent component TIC0. In this manner, we perform data‐driven estimation of a bespoke reaction coordinate for each CMP candidate along which we can drive umbrella sampling calculations to estimate the equilibrium free energy difference. The TICA analysis is conducted using the Deeptime Python library [88] employing pairwise distances between all α‐carbons as the molecular featurization. We present in Figure S3 a correlation analysis of the learned TIC0 coordinates with the radius of gyration $R_{g}$ of the most collapsed strand to illustrate that it serves as a physically interpretable reaction coordinate linking the triple helical and denatured states.

Umbrella sampling calculations were performed for each CMP candidate by distributing 42 uniformly spaced points along the range of the calculated TIC0 and harvesting from the temperature ramping runs the closest configuration to each point to serve as the initial simulation configuration. Independent molecular dynamics calculations at 320 K and 1 bar were conducted in each umbrella window employing the same simulation parameters detailed in Section 2.3 and applying harmonic restraining potentials in TIC0 with force constants of 1000 kJ/mol. We conducted a 100 ps equilibration run followed by a 40 ns production run. The biased simulations in TIC0 were conducted using a PYTORCH_MODEL collective variable in PLUMED 2.9.2 [56] enabled by the MLColvar package [89]. We recovered estimates of the unbiased potential of mean force (PMF) in TIC0 using the Weighted Histogram Analysis Method (WHAM) [90, 91], from which we computed the free energy difference ΔF between the triple helix and denatured states. In cases where we observed poor overlaps in the TIC0 histograms of neighboring umbrella windows, we augmented our umbrella sampling calculations with intermediate windows to ensure stable convergence of the WHAM calculations and recovery of smooth PMF profiles. Uncertainties in the PMF profiles were estimated using a bootstrapping procedure, in which we generated five resampled datasets by randomly selecting data points with replacement and computing the standard deviation across the resulting PMFs. Calculation of the converged PMF for a single CMP candidate sequence requires a total of approximately 1680 ns of enhanced sampling molecular dynamics simulation at a cost of approximately 1300 GPU‐hours. The simulation parameters and PLUMED files for the umbrella sampling calculations are provided in PLUMED‐NEST at www.plumed‐nest.org/eggs/25/013 [92].

### 2.7 Experimental Peptoid Synthesis and Characterization

#### 2.7.1 Materials

All solvents utilized for peptoid synthesis were sourced from Fisher or VWR and employed without additional purification. Milli‐Q water was consistently used throughout the experimental procedures. Rink amide resin (0.7–1.0 meq/g) and bromoacetic acid (BrAA) were from Chem‐Impex International, Inc. N,N‐diisopropylcarbodiimide (DIC), 4‐methylpiperidine (PIP), N‐Boc‐1,4‐butanediamine (*K*) and trifluoroacetic acid (TFA) were from Oakwood Chemical. β‐alanine tert‐butyl ester (*E*) hydrochloride was acquired from Ambeed, and *E* was deprotected according to a previous protocol prior to utilization [93].

#### 2.7.2 Synthesis of (EKGEKG)

Solid‐phase synthesis was conducted using procedures reported previously [93]. Rink amide resin (0.18 mmol) was swelled at in DMF for 10 mins. After filtration, the Fmoc groups on the resin were deprotected by adding 4 mL of 20% (v/v) 4‐PIP/DMF, agitating for 40 min, filtering, and washing with DMF five times. DMF washes consisted of the addition of 2 mL of DMF, followed by agitation for 1 min. An acylation reaction was then performed on the amino resin by adding 3 mL of 0.6 M BrAA in DMF and 2 mL of 50% (v/v) DIC/DMF. The mixture was agitated for 10 min at room temperature, filtered, drained and washed with DMF five times. Nucleophilic substitution occurred by adding 3 mL of 0.6 M primary amine monomers in NMP, followed by agitation for 10 min at room temperature. The monomer solution was filtered from the resin and washed with DMF five times. The acylation and substitution steps were repeated until the targeted peptoid (EK) was synthesized. 3 mL of 0.6 M Fmoc‐Gly‐OH in DMF and 2 mL of 50% (v/v) DIC/DMF were added to the EK resin, followed by 30 min agitation. The Fmoc group was removed by adding 4 mL of 20% (v/v) 4‐PIP/DMF and agitating for 40 min. The mixture was filtered and washed with DMF five times to get EKG. The EKG synthesis was iteratively performed six times to get (EKG)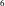 (i.e., (EKGEKG)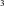).

#### 2.7.3 Purification

The peptoid product was cleaved by treating the resin beads with a 3 mL solution of TFA in water (95/5, v/v) for 30 min with agitation. The resulting solution was collected, and the TFA was evaporated under reduced pressure at 46 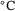. Subsequently, the crude product was dissolved in water and purified using reverse‐phase high‐performance liquid chromatography (HPLC). The purification procedure utilized a Waters 1525 system equipped with an XBridgeTM Prep C18 OBDTM column (10 μm, 19 mm × 100 mm). A linear gradient of acetonitrile in water 0% –10% (v/v) was applied for purification. All eluted fractions were combined and further confirmed by two gradients of acetonitrile in water 0‐10% and 5‐95% (v/v).

#### 2.7.4 Self‐Assembly of (EKGEKG)

A solution of 500 μM (EKGEKG)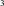 with 20 mM HEPES and 150 mM NaCl buffer was incubated at 80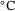 for 30 min. After cooling down to room temperature, the solution was placed at 4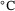 for 48 h.

#### 2.7.5 Scanning Electron Microscopy Characterization

Ex situ scanning electron microscopy (SEM) imaging was carried out on Thermofisher Apreo Low Vac 2S Scanning Electron Microscope. For the sample preparation, 1.5 μL of the self‐assembled peptoid was diluted in 100 μL D.I. water and deposited onto a 5 × 5 mm silicon wafer. Solution was removed by Whatman filter paper after a 15‐min interval. The instrument was operated at 5 kV voltage and 0.2 nA current.

## 3 Results

### 3.1 Active Learning Screen for Thermostable CMPs

We present the results of our computational active learning campaign in Figure 4. Commencing from an initial set of 26 randomly selected CMP candidates in Round 0, we conducted a 24‐round campaign in which a GPR surrogate model trained on atom pair fingerprints was coupled with batched BO to select eight new CMP candidates in each round. We initially employed a balanced exploit‐explore BO strategy employing a qEI trade‐off parameter of β = 0. After six consecutive rounds without observing any improvement in the top‐8 $T_{m}$, we switched in Round 18 to a pure exploit strategy by employing β = $-T_{m}^{\text{MAX}}$, where $T_{m}^{\text{MAX}}$ is the maximum $T_{m}$ value observed over all candidates to date. After ceasing to see any additional improvement in the top‐8 $T_{m}$ for four consecutive rounds, we terminated the active learning campaign after Round 24.

The active learning campaign considered a total of 218 CMP candidates (2.18% of the design space) and employed an aggregated total of 66.8 μs of simulation costing approximately 6400 GPU‐hours of compute. The top 16 CMP candidates with the highest $T_{m}$ values identified over the 24‐round active learning campaign are listed in Table 2. The top performing sequence, (EKGEKG)_3_ was identified in Round 8 and possesses a $T_{m}$ = (432 ± 8) K. An accounting of all 218 CMP candidates, detailing the active learning round in which they were selected and the calculated $T_{m}$ values, is provided in Table S1. An illustration of the evolution of the predictions of the GPR and BO over the course of each round of the active learning campaign are presented in Figures S4 and S5. In Figure S6, we present box plots of the absolute error between the GPR predictions and the subsequently calculated $T_{m}$ values in each round of the active learning campaign. As anticipated, we observe an improvement in GPR predictive accuracy as the campaign proceeds, attaining a mean absolute error (MAE) of 17.6 K over the terminal six rounds of the campaign.

**TABLE 2:** Top 16 CMP candidates identified the 24‐round active learning campaign.

Rank	CMP sequence	$T_{m}$ (K)	Round of discovery
	(x1‐y1‐G‐x2‐y2‐G)_3_		
1	(EKGEKG)_3_	432 ± 8	8
2	(EKGDKG)_3_	421 ± 5	19
3	(EKGEXG)_3_	411 ± 4	9
4	(EKGKEG)_3_	410 ± 5	8
5	(EXGEKG)_3_	410 ± 7	10
6	(EKGKVG)_3_	409 ± 4	1
7	(EKGDQG)_3_	408 ± 5	20
8	(KEGEKG)_3_	407 ± 4	7
9	(EKGEQG)_3_	406 ± 5	12
10	(KIGEKG)_3_	405 ± 5	6
11	(KEGKEG)_3_	405 ± 7	8
12	(KEGKIG)_3_	403 ± 4	3
13	(KIGKEG)_3_	401 ± 5	14
14	(KZGDKG)_3_	401 ± 5	17
15	(EKGEMG)_3_	400 ± 9	24
16	(EKGKYG)_3_	398 ± 5	24
Collagen	(PPGPPG)_3_	555 ± 8	—

Sequences of the peptoid hexamer repeat are reported using the one letter codes provided in Table 1. For the residues present in these 16 candidates, letters correspond to the peptoid counterpart of the amino acid sharing that one letter code, except for “Z”, which refers to the N‐*S*‐1‐phenylethyl side chain, and “X”, which refers to the aminoethyl side chain. For reference, we also report the $T_{m}$ of a natural collagen (PPGPPG)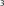 calculated using the same non‐equilibrium temperature ramping technique.

### 3.2 Composition Analysis of Thermostable CMPs

**FIGURE 5:**
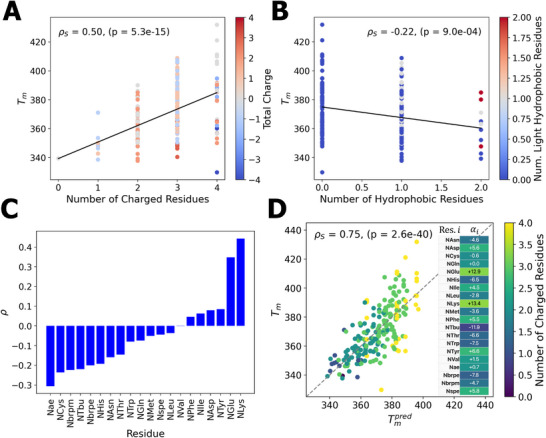
Composition‐thermostability analysis of the 218 CMPs considered in the active learning campaign. (A) The Spearman correlation coefficient between $T_{m}$ and the number of charged residues in the hexamer repeat is moderately strong and statistically significant $\rho_{S}$ = 0.50 (p = 5.3 × $10^{-15}$). We color each point by the total charge of the hexamer. (B) The Spearman correlation coefficient between $T_{m}$ and the average number of hydrophobic residues within the four guest residues in the hexamer repeat is weak but statistically significant $\rho_{S}$ = −0.22 (p = 9.0 × $10^{-4}$). We color each point by the number of light hydrophobic residues—NPhe, NLeu, NTyr, and NIle—and note that the negative correlation vanishes if we consider only these light hydrophobes. (C) Spearman correlation with $T_{m}$ of the frequency of each individual residue within the CMP. We note that this analysis is restricted to the 18 residues appearing within the 218 CMP candidates considered in the active learning screen. Long, charged residues like NLys and NGlu are positively correlated with $T_{m}$, while short positively charged residues like Nae are negatively correlated with $T_{m}$. While some bulky and hydrophobic residues seem to have a relative destabilizing effect on triple helices, the relatively lightweight NVal, NPhe, NIle, and NTyr seem to follow the opposite trend, suggesting a desirable zone of hydrophobic interactions within the triple helix. (D) A linear regression model to predict $T_{m}$ as a function of the count of each residue type within the heaxamer repeat possesses an RMSE = 12.2 K but a strong and statistically significant Spearman correlation coefficient $\rho_{S}$ = 0.75 (p = 2.6 × $10^{-40}$). The table reports the learned regression coefficients $\alpha_{i}$ for each count of residue i under the linear model specified in Equation (1).

The active learning screen furnished $T_{m}$ predictions for 218 CMP candidates, presenting a rich data set from which we seek to extract compositional determinants associated with high thermostability. We first sought to identify if the presence of charged residues mediating Coulombic interactions are correlated with stability of the triple helix. Electrostatic effects have been linked to triple‐helix stability, with studies showing that specific charge pairings, such as lysine and glutamate, can enhance stability [42]. Kessler et al. [19] similarly noted that charged peptoid residues modulate helix formation. As illustrated in Figure 5A, we observe a moderately positive and statistically significant Spearman correlation coefficient of $\rho_{S}$ = 0.50 (p = 5.3 × $10^{-15}$) between $T_{m}$ and the number of charged residues per hexamer repeat, although the spread in the number of charged residues is small and there is a high degree of scatter in the $T_{m}$ values. In Figure 5B, we probe the influence of the hydrophobicity of the guest residues upon thermal stability. We used a simple hydrophobicity proxy measurement by simply counting the number of NPhe, NIle, NLeu, NVal, NTrp, NTyr, Nbrpe, Nbrpm, Ntbu, and Nspe residues in each sequence. We observed a moderately negative and statistically significant Spearman correlation coefficient of $\rho_{S}$ = −0.22 (p = 9.0 × $10^{-4}$) between $T_{m}$ and the number of hydrophobic residues in the hexamer repeat. This modest negative trend is supported by host–guest CMP studies showing that inserting non‐polar residues such as leucine and phenylalanine tends to lower the melting temperature [94]. However, we caution against interpreting our negative correlation in isolation as evidence that hydrophobicity intrinsically destabilizes the helix, but must view this in light of the active learning campaign favoring charge‐rich sequences wherein incorporating a hydrophobic residue often replaced a charged side chain that was contributing a putative electrostatic stabilization.

To further dissect residue‐level contributions, we computed the Spearman correlation between the frequency of each individual residue within the CMP and the corresponding $T_{m}$ (Figure 5C). This analysis exposes that long, charged residues such as NLys and NGlu exhibit strong positive correlations with $T_{m}$, suggesting that their size and charge may facilitate favorable inter‐ or intra‐strand interactions (cf. Table 1). In contrast, short positively charged residues such as Nae showed a strong negative correlation with $T_{m}$, indicating that not all charged residues confer stabilizing effects, and that residue‐specific properties such as side chain length and flexibility are critical in resolving the subtleties of these interactions. While hydrophobic effects play an important role in triple helix stability, their contribution is nuanced. When hydrophobic residues are analyzed as a single group, no strong correlation with thermostability emerges. However, this masks opposing trends within the group: residues with smaller hydrophobic side chains—such as NIle, NVal, NPhe, and NTyr—tend to moderately enhance stability, possibly by promoting favorable close packing. In contrast, hydrophobic residues with large, bulky side chains—such as those containing *tert*‐butyl groups, indole rings, or bromine atoms—disrupt triple helical stability. This destabilization may be due to steric hindrance overriding the stabilizing hydrophobic interactions. These findings highlight that not all hydrophobic residues contribute equally to triple helix formation, and suggest that excessive steric bulk, despite its potential to rigidify peptoid backbones [95, 96], can be detrimental to overall structural integrity.

Motivated by the strong correlation of the frequencies of certain residues with thermostability, we constructed a simple linear regression model to predict $T_{m}$ as a function of the count of each residue type within the hexamer repeat of the form,

(1) $$\begin{array}{c} {\hat{T}}_{m}=c+\sum_{i}\alpha_{i}C_{i}, \end{array}$$

where $C_{i}$ is the count of residue type i within the hexamer repeat, $\alpha_{i}$ are the regression coefficients, c is the model intercept, and ${\hat{T}}_{m}$ is the predicted value of $T_{m}$. A least squares regression over the 218 CMPs with calculated $T_{m}$ values produced a model that, despite a root mean square error in $T_{m}$ prediction of RMSE = 12.2 K, achieved a strong and statistically significant Spearman correlation coefficient of $\rho_{S}$ = 0.75 (p = 2.6 × $10^{-40}$) (Figure 5D). The capacity of a simple linear regression model to predict the rank ordering of CMP stability indicates that composition alone is a reasonably good determinant of triple helix stability without appealing to sequence‐level correlations.

Taken together, these results underscore a critical role for Coulombic interactions and certain hydrophobic interactions in stabilizing peptoid triple helices. The observed correlations between $T_{m}$ and the presence of charged residues suggest that interstrand electrostatics and hydrophobicity promote triple helix stability, as has been observed in certain synthetic collagen‐mimetic peptides [96, 97]. These interactions are especially important in the context of peptoids, which lack the hydrogen bonding and proline‐ or hydroxyproline‐induced stereochemical preorganization of the backbone into extended polyproline‐II‐like helices as in natural collagen [18, 98, 99]. Without these intrinsic structural cues in the backbone, it falls to the chemistry and positioning of the peptoid side chains to promote single‐strand polyproline‐II helix‐like conformations and stabilize the triple helix bundle.

### 3.3 Thermodynamic Stability of Top Performing CMPs

**FIGURE 6:**
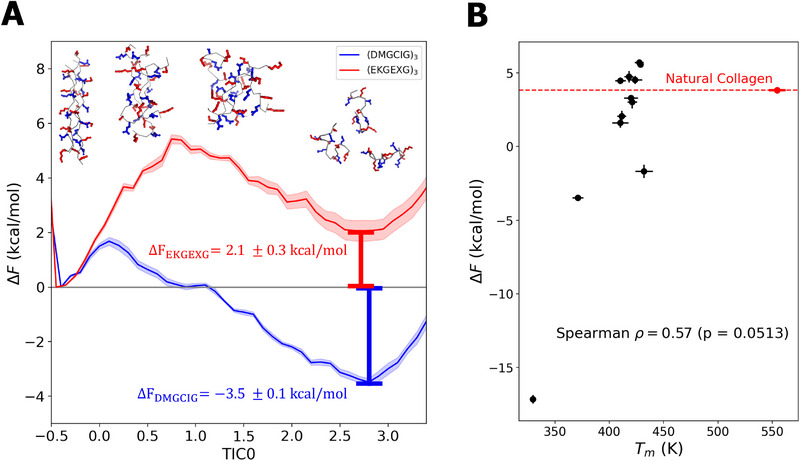
Thermodynamic stability calculations of the top‐ranked thermostable CMPs identified in the active learning screen. (A) Potential of mean force curves estimated by umbrella sampling for the (EKGEXG)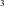 sequence that was ranked #3 in our active learning screen (red) and the (DMGCIG)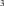 sequence that was ranked #97 (blue). Calculations are performed in the dominant time‐lagged independent component TIC0 estimated from a denaturation trajectory that serves as a reaction coordinate connecting the native collagen triple helix and a melted disordered aggregate. The free energy of denaturation ΔF was estimated as the free energy difference between the low‐TIC0 local minimum containing the triple helix state and the high‐TIC0 local minimum containing the denatured state. The arbitrary zero of the PMF curves was set to the free energy of the triple helix state, but is immaterial since only free energy differences are meaningful. Representative structures of the (EKGEXG)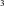 system at various values of TIC0 are superposed on the plot to illustrate the structural transitions along TIC0. (B) Comparison of non‐equilibrium temperature ramping $T_{m}$ stability estimates with equilibrium free energy differences ΔF at 320 K for the 13 CMP candidate sequences in Table 3. A moderately strong and statistically significant Spearman correlation coefficient $\rho_{S}$ = 0.57 (p = 0.0513) indicate that $T_{m}$ is a satisfactory and inexpensive measure of thermostability. We omit from this calculation the natural collagen variant indicated by the red point that stands as an outlier with an artificially elevated $T_{m}$ value, possibly attributable to kinetic trapping within the non‐equilibrium temperature ramping calculations of the highly constrained (PPGPPG)_3_ backbone containing multiple proline residues. The five sequences lying above the horizontal dashed red line possess ΔF values exceeding that of the natural collagen.

The temperature ramping simulation estimates of $T_{m}$ were designed to provide a computationally efficient but approximate and non‐equilibrium estimation of relative CMP thermostability that enabled high throughput within the virtual screen. After completion of the active learning campaign, we subjected the top candidates to more rigorous enhanced sampling free energy calculations of stability. Specifically, we estimated the potential of mean force curve at 320 K along the dominant time‐lagged independent component TIC0 estimated from a denaturation trajectory as a convenient reaction coordinate connecting the native collagen triple helix and a melted disordered aggregate (Section 2.6). We present in Figure 6A potentials of mean force for two representative CMPs and the corresponding free energies of denaturation ΔF of the collagen triple helix to the melted state. Large positive values of ΔF correspond to more stable collagen triple helix conformations. The thermostable CMP candidate (EKGEXG)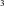 that was ranked #3 in our active learning screen possesses a moderately positive ΔF = (2.1 ± 0.3) kcal/mol (red curve), whereas the unstable (DMGCIG)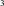 sequence that was ranked #97 in our screen possesses a moderately negative ΔF = (−3.5 ± 0.1) kcal/mol (blue curve). Uncertainties in ΔF are propagated from the uncertainties in the underlying potentials of mean force by bootstrapping.

We report in Table 3 the calculated ΔF values for a total of 13 CMP candidates. We consider the top five most thermostable CMPs identified in our active learning screen—(EKGEKG)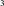, (EKGDKG)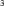, (EKGEXG)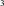, (EKGKEG)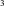, (EXGEKG)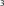 (#1–5)—as well as an intermediate ranked—(DMGCIG)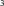 (#97)—and lowest ranked—(EDGDDG)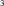 (#218)—candidates as negative controls. As a positive control, we consider a natural collagen variant—(PPGPPG)
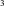
—which, due to the special character of the G and P residues, is classified as both a peptide and peptoid. Finally, inspired by the observation in our temperature ramping calculations that triple helix collapse tends to begins by the fraying of the terminal residues and the knowledge that proline residues in natural collagen promote preorganization of the backbone into extended polyproline‐II‐like helices that stabilize the collagen triple helix [18, 98, 99], we performed rational modifications of the top five‐ranked CMPs by eliminating one hexamer repeat and replacing it with two flanking PPG motifs—PPG(EKGEKG)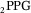, PPG(EKGDKG)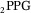, PPG(EKGEXG)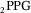, PPG(EKGKEG)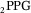, PPG(EXGEKG)
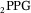
—under the hypothesis that this modification may help to further boost the thermostability.

**TABLE 3:** Thermodynamic stability of the top ranked CMP candidates identified in the active learning screen.

Class	Sequence	Rank	$T_{m}$ (K)	ΔF (kcal/mol)
Screen	(EKGEKG) 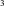	1	432 ± 8	−1.7 ± 0.5
	(EKGDKG) 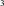	2	421 ± 5	3.0 ± 0.4
	(EKGEXG) 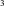	3	411 ± 4	2.1 ± 0.4
	(EKGKEG) 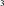	4	410 ± 5	**4.5 ± 0.2**
	(EXGEKG) 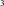	5	410 ± 7	1.6 ± 0.3
	(DMGCIG) 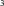	97	371 ± 5	−3.5 ± 0.2
	(EDGDDG) 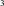	218	330 ± 1	−17.1 ± 0.3
Engineered	PPG(EKGEKG) 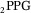	(1)	424 ± 7	4.5 ± 0.3
	PPG(EKGDKG) 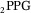	(2)	420 ± 6	3.3 ± 0.2
	PPG(EKGEXG) 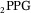	(3)	418 ± 4	4.8 ± 0.4
	PPG(EKGKEG) 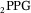	(4)	429 ± 2	5.6 ± 0.2
	PPG(EXGEKG) 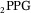	(5)	428 ± 4	**5.7 ± 0.2**
Collagen	(PPGPPG) 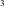	—	555 ± 8	**3.8 ± 0.2**

*Note*: The free energy of denaturation of the collagen triple helix ΔF provides a quantitative measure of the thermodynamic stability of the CMP candidate to exist in this state, with large positive values corresponding to higher stability candidates. We also report the melting temperatures $T_{m}$ computed by non‐equilibrium temperature ramping calculations over the course of the active learning screen. The seven candidates in the *Screen* class correspond to the top five CMPs with the highest $T_{m}$ values identified by the active learning screen (#1–5), along with intermediate ranked (#97) and lowest ranked (#218) negative controls. The five candidates in the *Engineered* class represent engineered variants of the top five CMPs from the active learning screen in which a double hexamer repeat of the sequence is flanked by PPG motifs. Ranks of the parent sequence are reported in brackets. The final *Collagen* class presents a native collagen positive control. The ΔF values of the most stable candidate in each class is indicated in **bold** and those with stabilities exceeding that of natural collagen are colored red.

The ΔF and $T_{m}$ values for the 13 sequences in Table 3 are presented graphically in the scatterplot in Figure 6B that exposes a strong positive correlation between these two estimates of CMP stability. Only natural collagen stands as an outlier, possessing an anomalously high $T_{m}$ value relative to its ΔF. We suggest that the $T_{m}$ value may have been artificially elevated in our non‐equilibrium temperature ramping calculations due to the highly constrained nature of the proline‐rich (PPGPPG)_3_ backbone that may have promoted kinetic trapping of the triple helix configuration. Dropping this point as an outlier, the remaining 12 points possess a moderately strong and statistically significant Spearman correlation coefficient $\rho_{S}$ = 0.57 (p = 0.0513), which provides a post hoc validation of our use of $T_{m}$ as a proxy measure of CMP thermostability within the active learning screen that is that is approximately seven‐fold less expensive to compute than ΔF.

Analyzing the ΔF results reveals that one of the top‐five ranked sequences in the active learning screen—(EKGKEG)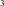 (#4)—is predicted to be 0.7 kcal/mol more stable than the natural collagen variant (PPGPPG)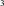 (Table 3). For computational expediency, we study relatively short 18‐mer peptoid sequences, whereas natural collagen chains can easily exceed 1000 residues [100], so it is more informative to frame this ostensibly small absolute value as a rather sizable stability increase of 0.04 kcal/mol.residue on a per residue basis or a 18% increase on a relative basis. As detailed in Section 3.2, the presence of long, charged side chains can promote strong inter‐strand electrostatic interactions that compensate for the absence of backbone rigidity and strongly stabilize the collagen triple helix. The remaining four top‐five $T_{m}$ sequences did not, however, surpass the ΔF of the natural collagen variant, and the two low‐ranked negative controls were verified to be highly unstable. These trends reflects the approximate nature of $T_{m}$ as a predictor of thermostability as graphically evinced by the scatter in Figure 6B: it can serve as a good approximate estimator of stability, but is challenged to resolve the finer grained differences between candidate sequences that can only be exposed by more accurate stability calculations.

Turning to the rationally engineered sequences, we observe that capping two hexamer repeats with flanking PPG motifs leads to a stability boost ranging from approximately 1–6 kcal/mol. This modification resulted in four of the five engineered sequences possessing superior ΔF values relative to the natural collagen variant, with the most stable sequence PPG(EXGEKG)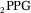 possessing a 1.9 kcal/mol stability elevation, corresponding to a substantial increase of 0.10 kcal/mol.residue on a per residue basis or 50% on a relative basis. The combination of charged and certain hydophobic residues within the hexamer repeat again appear to promote stabilizing inter‐strand electrostatic and hydrophobic interactions and the flanking PPG motifs appear to convey a stability benefit by protecting the termini against fraying by introducing backbone rigidity and helical preorganization to promote stronger hydrogen bonding and steric compatibility.

### 3.4 Experimental Testing

**FIGURE 7:**
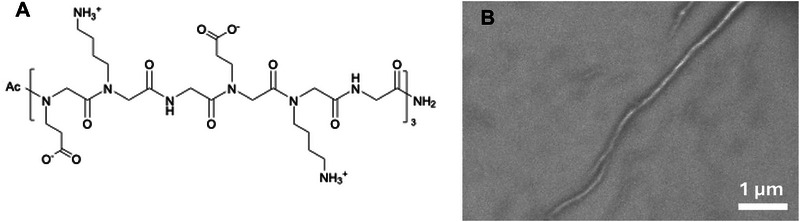
Experimental synthesis, assembly, and characterization of the predicted high‐performing peptoid (EKGEKG)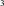 identified in the computational screen. (A) Chemical structure of the peptoid (EKGEKG)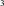. (B) SEM image of the fibril‐like bundles produced by self‐assembly of these peptoids. The fiber lengths are in excess of 10 μm, while the widths are sub‐nanometer, indicating the likelihood of individual stable triple‐helical bundles rather than higher‐order supramolecular assemblies.

We next sought to experimentally test the capability of the computational screen to identify peptoid sequences that assemble into collagen‐like triple helices. Both the non‐equilibrium $T_{m}$ and more rigorous equilibrium ΔF computational measures of stability commenced from a pre‐assembled triple helix and assessed the stability with respect to denaturation of the quaternary structure. The experimental tests, in contrast, assess the capability of the strands to spontaneously self‐assemble into the triple helix, representing a more stringent assessment of both assembly and stability. Furthermore, while the computations always commenced from a right‐handed triple helix corresponding to that of natural collagen, the achiral nature of peptoid chains mean that we expect no preference for left‐ or right‐handed triple helical assemblies and the helices to exist as a statistical mixture of the two supramolecular chiralities. We elected to synthesize and self‐assemble the peptoid sequence (EKGEKG)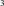, which is one of the top five most thermostable CMPs identified by our active learning screen (Table 2). Synthesis and purification was conducted using previously reported procedures [93]. Figure S7 illustrates our confirmation of a successful synthesis and purification via ultra‐performance liquid chromatography‐mass spectrometry (UPLC‐MS). After synthesis, purification, and self‐assembly (Section 2.7), imaging of the resulting aggregate using scanning electron microscopy (SEM) resolved a fibril‐like bundle consistent with a collagen‐like triple helix (Figure 7). The fibril lengths are in excess of 10 μm, whereas the widths are on the sub‐nanometer scale, indicating the likelihood of individual stable triple‐helical bundles rather than higher‐order structures involving further aggregation. The SEM results provide support for the assembly of the (EKGEKG)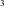 peptoids into collagen‐like triple helices, but we acknowledge that more detailed characterizations such as cryo‐electron microscopy would be required to probe the assemblies at the molecular scale and validate the presence of the triple helical structure within the observed fibrils.

## 4 Conclusions

This work introduces a computational active learning strategy for the design and discovery of peptoid‐based CMPs capable of forming stable triple‐helical structures. Using a combination of molecular dynamics simulations, Gaussian process regression, and batched BO, we identified a number of hexamer repeat sequences predicted to possess highly stable triple helix conformations. Our active learning protocol achieved these results by sampling only 2.18% of a 10 000‐member design space, substantially reducing the computational effort of a random or exhaustive search while uncovering candidates that might elude traditional, intuition‐driven approaches. The rank‐ordered list of predicted thermostable CMPs offers a computationally efficient filtration of the design space, and compositional analysis of the results of the screen reveals design principles underpinning peptoid triple helix stability, including the strategic placement of charged and light hydrophobic residues to compensate for the absence of proline‐driven backbone restriction. Subjecting the top CMPs to more rigorous scrutiny under enhanced sampling free energy calculations identifies (EKGKEG)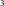 as a peptoid‐based CMP with 18% higher thermostability that a (PPGPPG)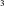 natural collagen variant. We further show that this stability can be further elevated by adding flanking PPG motifs to induce additional helicity into the strands, resulting in the prediction of PPG(EXGEKG)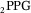 as a rationally engineered variant with 50% higher stability than the natural collagen variant. Experimental synthesis and characterization of one of the top‐performing sequences (EKGEKG)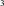 identified by the computational screen resolved fibril‐like bundles under SEM imaging that are consistent with collagen‐like triple helices.

Taken together, this work presents a data‐driven computational screen to engineer quaternary structure into peptoid chains, advancing the potential for control of the structure and function of these synthetic biomimetic polymers. The computational screening methodology can be generalized to other biomolecular and macromolecular systems where stability, chemical diversity, and functionality are key design goals. In future work, we would like to expand this analysis to other peptoid‐based materials with other functionalities such as surface binding [101], structure formation [102], or enzymatic catalysis [103]. We also see opportunities for technical innovations in the active learning campaign by incorporating ideas from multi‐objective BO to conduct co‐optimization of (possibly competing) functional goals [104] and multi‐fidelity BO to incorporate data from multiple computational and experimental screens at various degrees of resolution, cost, and fidelity [105]. Finally, we incorporated domain knowledge into our engineering protocol in a relatively weak manner by appealing to observations of the role of terminal fraying in denaturation mechanisms and the role of PPG groups as natural helix‐promoters, but envisage the exploration of other terminal capping motifs and specific residue–residue interactions as well as more rigorous means to incorporate priors into the engineering campaign as inductive biases to improve learning and accelerate the active learning search [106].

## Conflicts of Interest

Andrew L. Ferguson is a co‐founder and consultant of Evozyne, Inc. and a co‐author of US Patent Applications 16/887,710 and 17/642,582, US Provisional Patent Applications 62/853,919, 62/900,420, 63/314,898, 63/479,378, 63/521,617, and 63/669,836, and International Patent Applications PCT/US2020/035206, PCT/US2020/050466, and PCT/US24/10805.

## Supporting information


**Supporting File 1**: marc70225‐sup‐0001‐SuppMat.pdf.

**Supporting File 2**: marc70225‐sup‐0002‐TableS1.csv.

## Data Availability

Jupyter notebooks implementing all computational components of the active learning workflow are made freely available at https://github.com/Ferg‐Lab/cmp. The simulation parameters and PLUMED files for the enhanced sampling calculations are provided in PLUMED‐NEST at www.plumed‐nest.org/eggs/25/013.
