# Supplementary material for: Data‐Driven Engineering of Thermostable Collagen‐Mimetic Peptoid Triple Helices
Source: Macromol Rapid Commun. 2026 Feb 24;47(8):e00917. doi: 10.1002/marc.202500917 (PMC13087863; doi:10.1002/marc.202500917)
Supplement: Supplementary file 1 — Supporting File 1: marc70225‐sup‐0001‐SuppMat.pdf. [file MARC-47-e00917-s001.pdf]

SUPPORTING INFORMATION:
Data-Driven Engineering of Thermostable Collagen-Mimetic Peptoid Triple Helices

Alex Berlaga
Department of Chemistry, University of Chicago, Chicago, IL, 60637, USA
berlaga@uchicago.edu

Renyu Zheng
Department of Chemical Engineering, University of Washington, Seattle, WA, 98195, USA
renyuz2@uw.edu

Zeqian Zhang
Department of Chemistry, University of Washington, Seattle, WA, 98195, USA
francisz@uw.edu

Junhee Lee
Pritzker School of Molecular Engineering, University of Chicago, Chicago, IL, 60637, USA
jlee768@uchicago.edu

Diya Gandhi
Pritzker School of Molecular Engineering, University of Chicago, Chicago, IL, 60637, USA
diya.adiga.gandhi@gmail.com

Chun-Long Chen
Physical Sciences Division, Pacific Northwest National Laboratory, Richland, WA, 99352, USA
chunlong.chen@pnnl.gov

Andrew L. Ferguson*
Pritzker School of Molecular Engineering, University of Chicago, Chicago, IL, 60637, USA
Department of Chemistry, University of Chicago, Chicago, IL, 60637, USA
andrewferguson@uchicago.edu

**Author to whom correspondence should be addressed.*

S1 Supplementary Figures

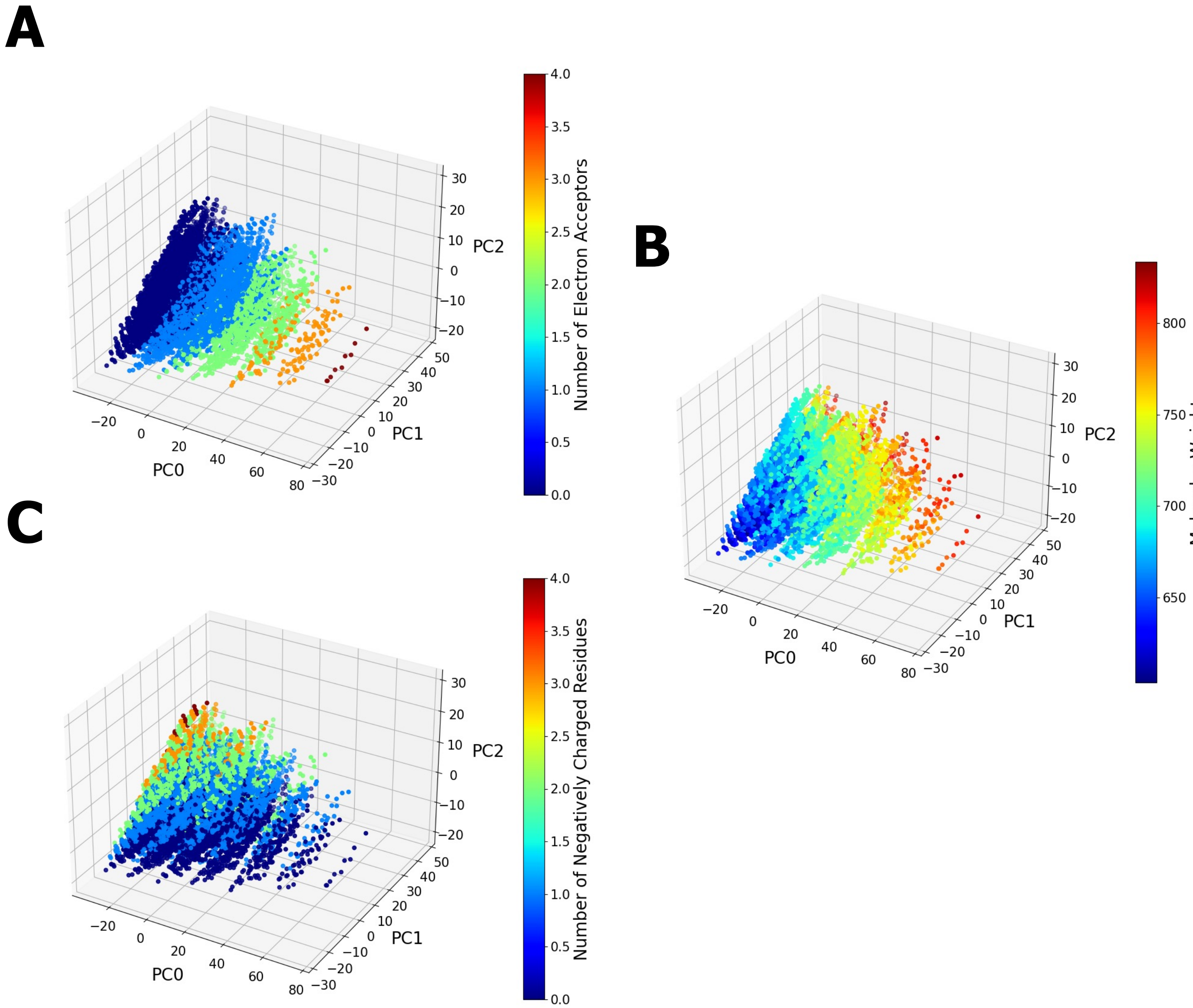

Figure S1: Projection of the 10,000 candidate CMPs into the top three principal components (PC0, PC1, PC2). Scatterplots are colored by candidate physical variables that expose strong correlations with the leading PCs. (A) PC0 is correlated with the number of electron acceptors adjacent to a peptoid carbonyl, characterized by the presence of NLys residues in the first and fourth positions and Nspe, Nbrpe, or Nbrpm residues in the second and fifth positions. Interestingly, these residues may interact as electrophiles in n-π^* or nucleophile-electrophile interactions with the peptoid carbonyl oxygen. (B) PC1 is well correlated with the molecular weight of the hexamer. (C) PC2 is correlated with the number of negatively charged residues.

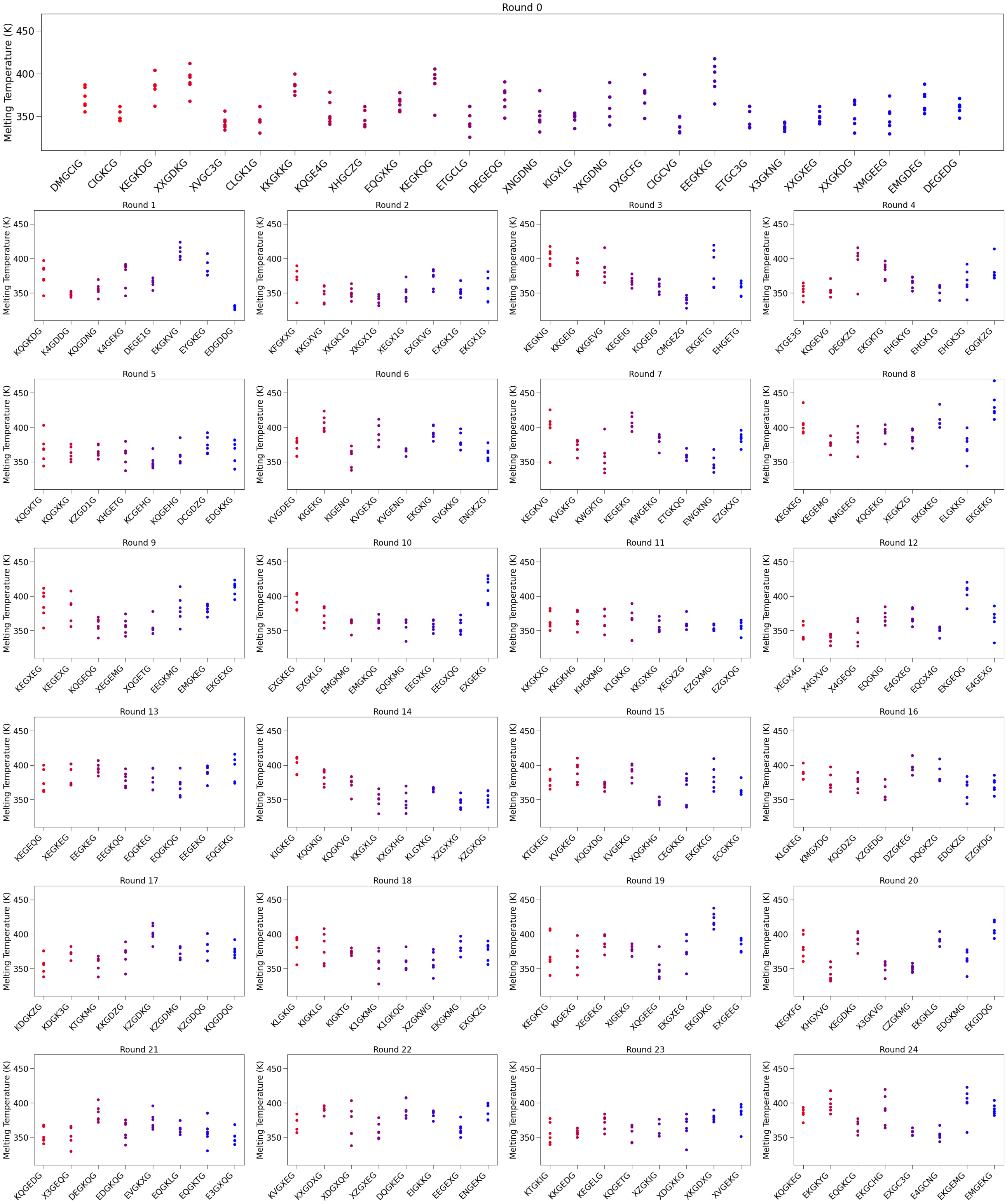

Figure S2: Graphical reporting of the CMP hexamer repeats $(x1\text{-}y1\text{-}Gly\text{-}x2\text{-}y2\text{-}Gly)_3$ considered in each round of the active learning campaign and the attendant T_m values estimated by temperature-ramping simulations. The initial round (Round 0) comprises 26 randomly selected CMP candidates to seed the active learning search, while all subsequent rounds comprise eight CMP candidates selected by batched Bayesian optimization using the qEI acquisition function. Six replicate simulations are conducted for each peptoid in each round to quantify uncertainties in T_m.

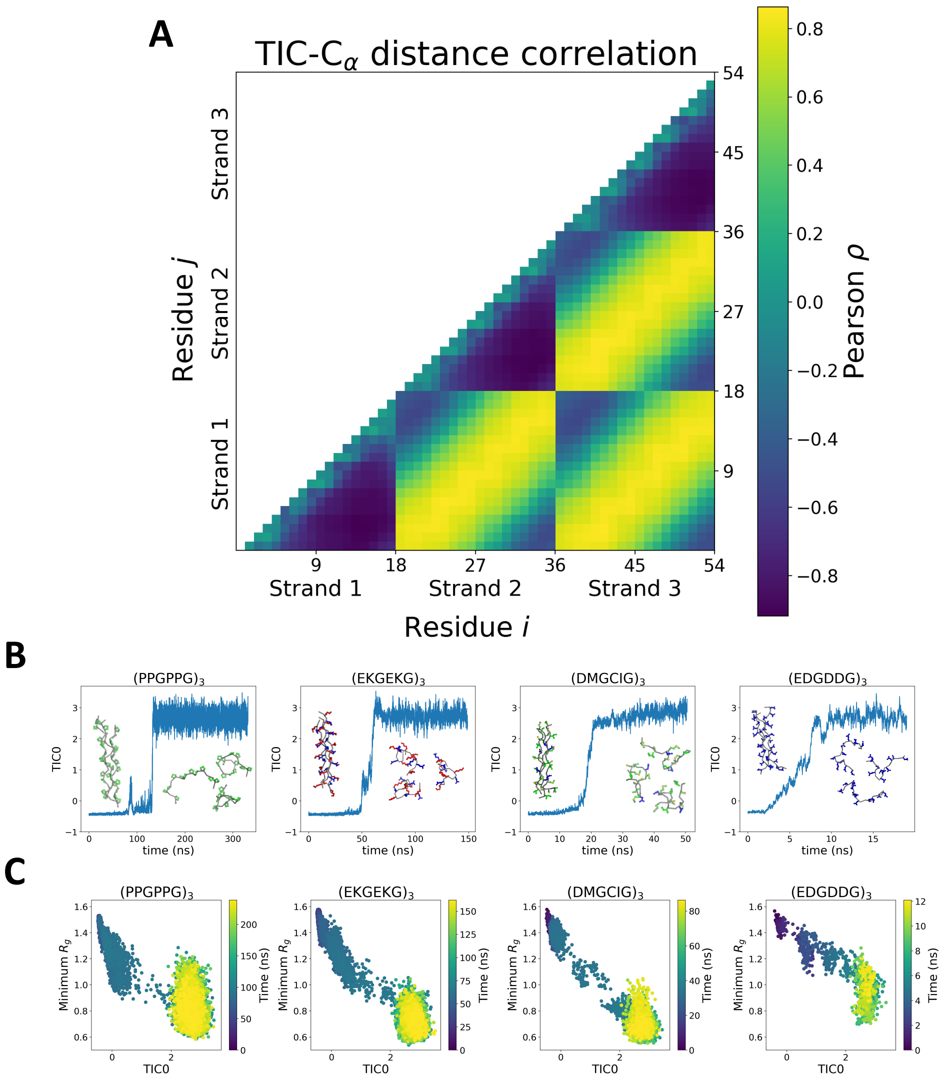

Figure S3: Data-driven determination of a reaction coordinate for umbrella sampling calculations between the native collagen triple helix and melted disordered aggregate using time-lagged independent component analysis (TICA) of the temperature ramping simulations. (A) Pearson correlation coefficients ρ of each C_α pairwise distance with the leading TICA component TIC0 for a concatenated trajectory of the natural collagen fragment $(PPGPPG)_3$ and the three CMP candidates $(EKGEKG)_3$, $(DMGCIG)_3$, and $(EDGDDG)_3$. Since there are three identical 18-mer CMP strands in the initial collagen triple helix, the α-carbon at position i on the first strand corresponds to equivalently positioned α-carbons at positions $(i{+}18)$ and $(i{+}36)$ on the second and third strands, respectively. Increasing TIC0 values are correlated with decreasing intramolecular α-carbon distances corresponding to collapse of each individual strand (blue) and increasing intermolecular α-carbon distances corresponding to separation of the strands (yellow). (B) Evolution of the TIC0 collective variable for natural collagen fragment $(PPGPPG)_3$ and three CMP candidates $(EKGEKG)_3$, $(DMGCIG)_3$, and $(EDGDDG)_3$. In each case the TIC0 exhibits a large jump at the point at which denaturation of the triple helix is observed. We simulate each system 2.5× past the time at which denaturation occurred. (C) Plot of the TIC0 collective variable against the radius of gyration R_g of the most collapsed strand that we use as a criterion to determine T_m in our non-equilibrium temperature ramping calculations for the same four CMP candidates. Comparing with panel B, the coincidence of the precipitous drop in minimum R_g and sharp increase in TIC0 supports the utility of both TIC0 and minimum R_g as metrics to diagnose denaturation of the collagen triple helix state.

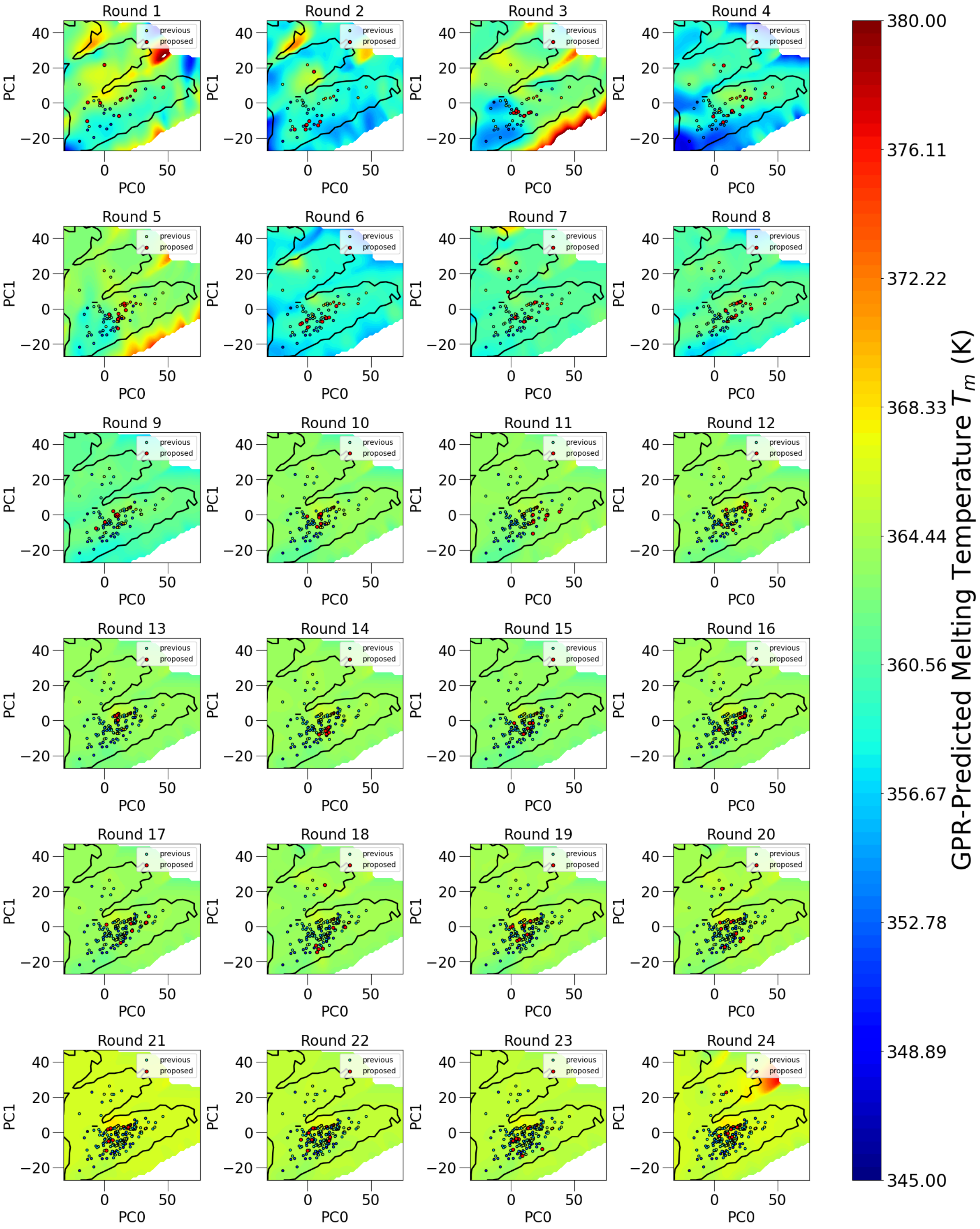

Figure S4: GPR predictions of melting temperature over CMP candidate space over the course of the 24-round active learning campaign. We illustrate the GPR predictions over a 2D latent space constructed in the top two principal components (PC0, PC1) of the atom pair featurization. The black contour encompasses the region within this projection containing all 10,000 CMP candidates in our design space. In each panel, we illustrate by red points the location in the latent space the eight CMP candidates selected by batched BO under a qEI acquisition function. The other points correspond to CMPs with T_m estimates calculated in previous active learning rounds and which are colored by T_m according to the colorbar on the right of the figure. The colored contour plot over the latent space is a kernel density smoothing of the GPR predicted T_m values for all CMP candidates not yet selected for evaluation.

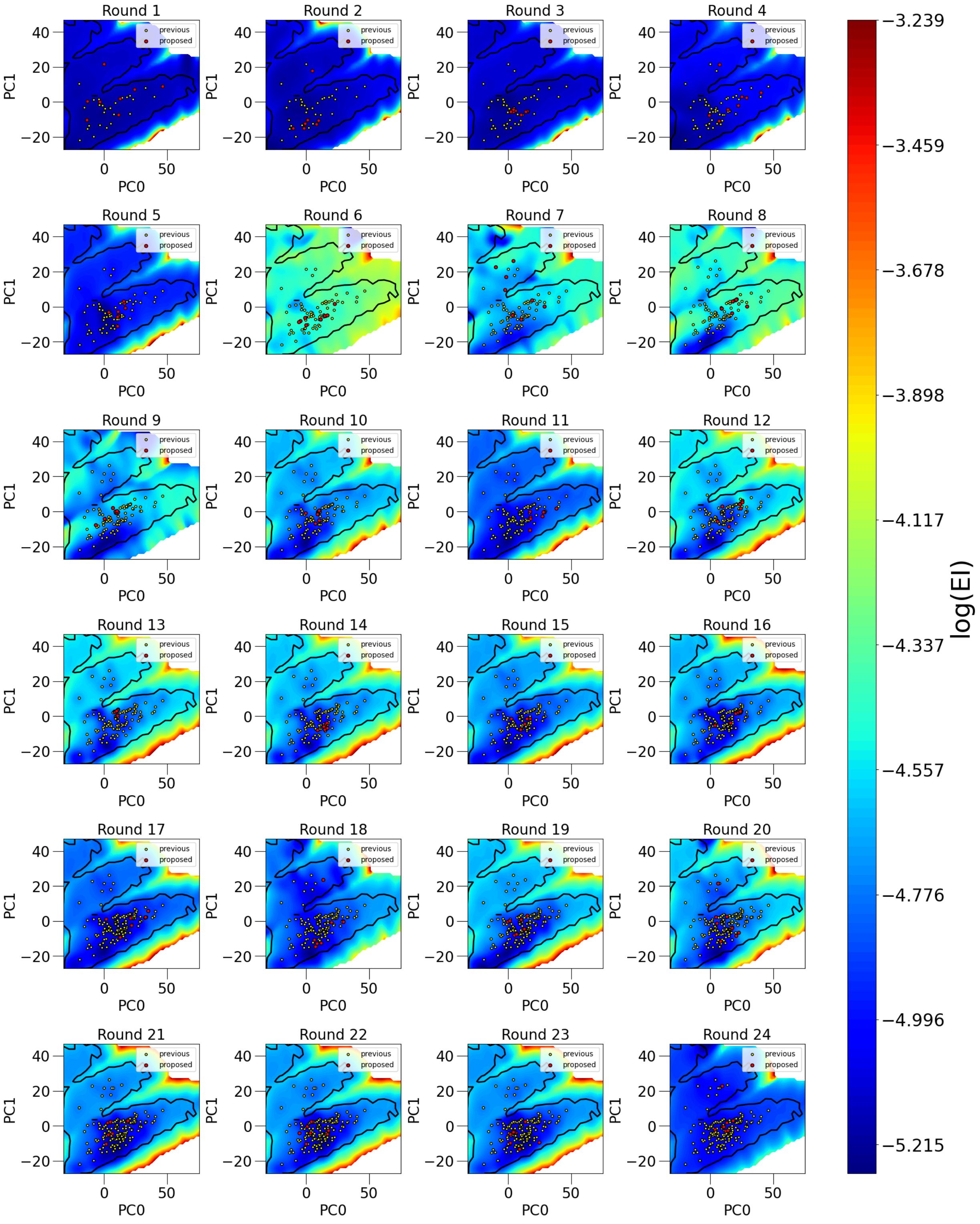

Figure S5: Batched BO selection of CMP candidates over the course of the 24-round active learning campaign. We illustrate the BO selections over a 2D latent space constructed in the top two principal components (PC0, PC1) of the atom pair featurization. The black contour encompasses the region within this projection containing all 10,000 CMP candidates in our design space. In each panel, we illustrate by red points the location in the latent space the eight CMP candidates selected by batched BO under a qEI acquisition function, and by yellow points the candidates subjected to T_m calculations over all previous rounds. The colored contour plot over the latent space is a kernel density smoothing of the EI acquisition function values for all CMP candidates not yet selected for evaluation.

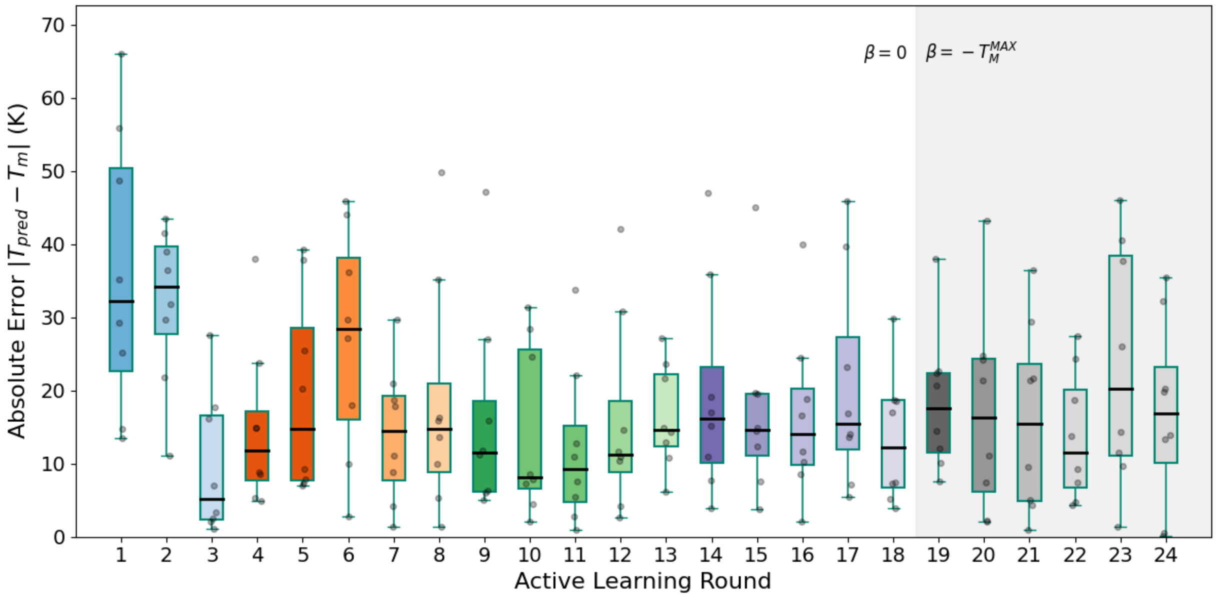

Figure S6: Boxplot distribution of the absolute prediction errors ($|T_{pred} - T_m|$) across 23 active learning rounds. Each box represents the error distribution for candidates selected in that specific round. Black points represent individual candidate residuals, while the horizontal bars indicate the median absolute error (MAE). As anticipated, we observe an improvement in GPR predictive accuracy as the active learning campaign proceeds, attaining a mean absolute error (MAE) of 17.6 K over the terminal six rounds of the campaign. The GPR accuracy oscillates in the early rounds but plateaus around Round 8, coinciding with the observation of the maximum true T_m.

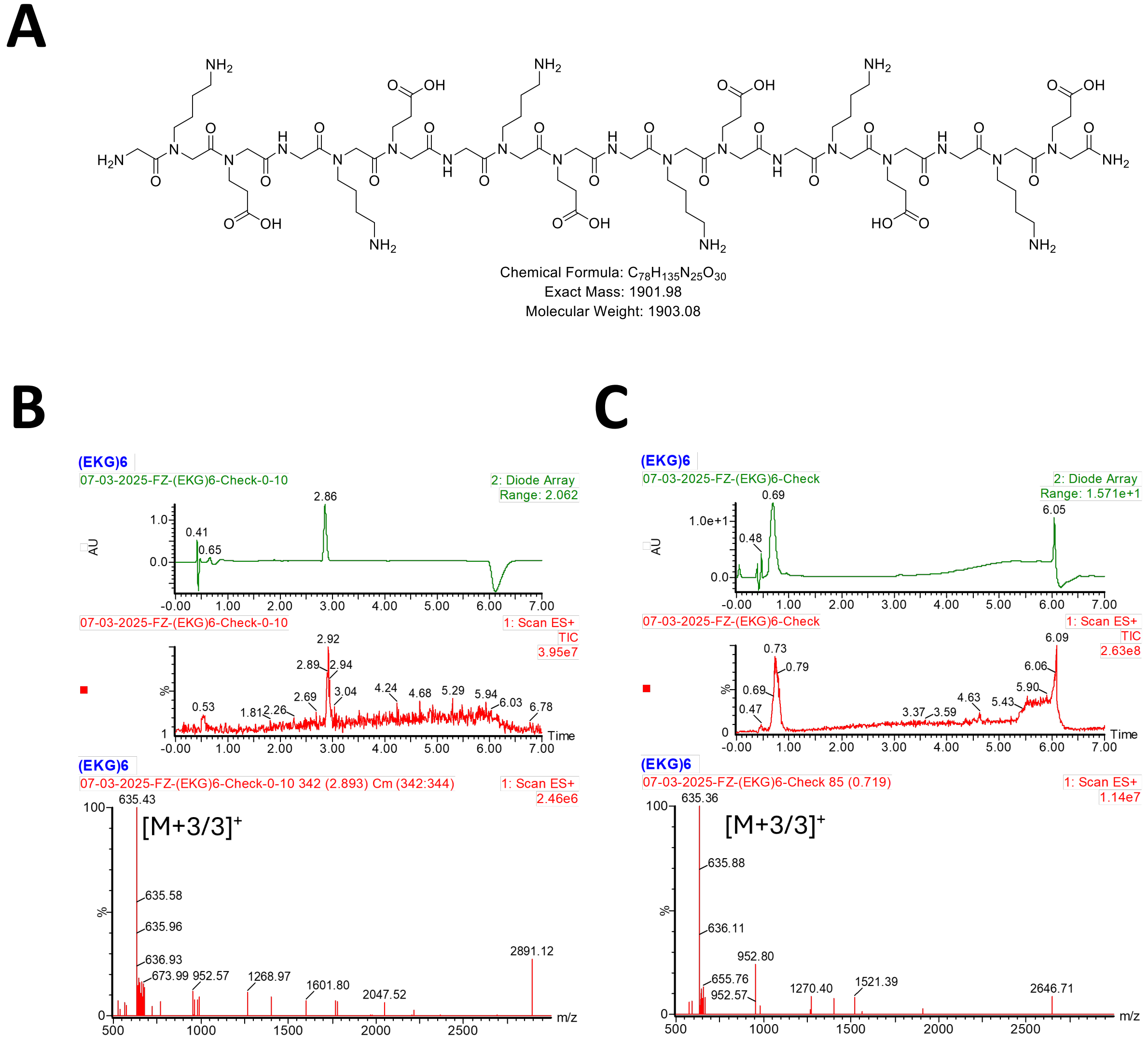

Figure S7: UPLC-MS data of HPLC-purified peptoid $(EKGEKG)_3$. (A) Molecular structure of $(EKGEKG)_3$. (B) UPLC-MS of $(EKGEKG)_3$ obtained with a gradient of acetonitrile from 0-10%. (C). UPLC-MS of $(EKGEKG)_3$ obtained with a gradient of acetonitrile from 5-95%.
